# Roquefort Cheese Proteins Inhibit *Chlamydia pneumoniae* Propagation and LPS-Induced Leukocyte Migration

**DOI:** 10.1155/2013/140591

**Published:** 2013-04-28

**Authors:** Ivan M. Petyaev, Naylia A. Zigangirova, Natalie V. Kobets, Valery Tsibezov, Lydia N. Kapotina, Elena D. Fedina, Yuriy K. Bashmakov

**Affiliations:** ^1^Lycotec Ltd. Granta Park Campus, Cambridge CB21 6GP, UK; ^2^Gamaleya Institute of Epidemiology and Microbiology, Ministry of Health, 18 Gamaleya Street, Moscow 123098, Russia

## Abstract

Inflammation in atherosclerosis, which could be associated with some subclinical infections such as *C. pneumoniae*, is one of the key factors responsible for the development of clinical complications of this disease. We report that a proprietary protein extract isolated from Roquefort cheese inhibits the propagation of *C. pneumoniae* in a human HL cell line in a dose-dependent manner, as revealed by the immunofluorescence analysis. These changes were accompanied by a significant reduction in the infective progeny formation over the protein extract range of 0.12–0.5 **μ**g/mL. Moreover, short term feeding of mice with Roquefort cheese (twice, 10 mg per mouse with an interval of 24 hours) led to the inhibition of the migration of peritoneal leukocytes caused by intraperitoneal injection of *E. coli* lipopolysaccharide. These changes were complemented by a reduction in neutrophil count and a relative increase in peritoneal macrophages, suggesting that ingestion of Roquefort could promote regenerative processes at the site of inflammation. The ability of this protein to inhibit propagation of *Chlamydia* infection, as well as the anti-inflammatory and proregenerative effects of Roquefort itself, may contribute to the low prevalence of cardiovascular mortality in France where consumption of fungal fermented cheeses is the highest in the world.

## 1. Introduction

An epidemiological link between the reduction of cardiovascular mortality and moderate wine consumption in French and Mediterranean adults, known as the “French paradox” [1], has attracted the steady interest of medicobiological researchers over the past two decades. It has been proven in numerous clinical and experimental studies that common constituents of red wine—resveratrol and other polyphenols—have multiple health benefits resulting from their anti-inflammatory [2], anticarcinogenic [3], and antiatherogenic [4] properties and thereby contribute to the occurrence of the “French paradox.” The molecular mechanism behind the biological activity of dietary polyphenols has been extensively reviewed [5] and is believed to be mediated by sirtuins, a family of NAD^+^-dependent histone deacetylases [6].

Thorough ongoing research into the matter has led to the gradual realization that there are other dietary and nondietary determinants contributing to reduced cardiovascular mortality in the French and Mediterranean populations. In particular, higher consumption of omega-3 fatty acid [7], flavonoids [8], and dietary fiber [9] may also contribute to the mechanisms of the “French paradox.”

Cheese and cheese-containing products are prominent ingredients of the Mediterranean diet. It has been assumed that high cheese consumption may somehow contribute to favorable changes in lipid profile and reduced risk of cardiovascular disease [10]. Indeed, the varieties of blue-veined and other fungal fermented cheeses, which are a trademark of French culinary culture, may possess some measurable health benefits due to the presence of numerous functional substances in their core. Leading commercial brands of blue cheese have been shown to contain short-chain fatty acids, methyl ketones, and secondary alcohols [11]. Assessment of the microbial population in blue cheese reveals that *Penicillium roqueforti, Penicillium glaucum*, and *Geotrichum candidum* are three major distinguishable fungi, while *Lactocococcus lactis, Lactococcus garvieae, *and* Lactococcus raffinolactis *can be identified in blue cheese specimens during different stages of ripening [12]. *P. roqueforti *metabolites in particular show a wide range of pharmacological activity. Andrastins A, B, C, and D are consistently produced in blue-veined cheese and are potent inhibitors of farnesyltransferase, a major enzyme of cholesterol biosynthesis [13]. Andrastin A is also known to display strong antitumor properties [13]. Other substances, including roquefortine, a compound with some neurotoxic properties, constrain Gram-positive bacterial growth by inhibiting cytochrome P-450 [14]. The biological activity of metabolites produced by other fungi has yet to be studied.

In the present paper we report that Roquefort cheese extract inhibits propagation of *C. pneumoniae *in cultured cell line,while Roquefort feeding attenuates the LPS-induced migratory response of peritoneal leukocytes and causes significant changes in immune cell subpopulations.

## 2. Materials and Methods

### 2.1. Reagents and Organisms

All reagents were from Sigma-Aldrich unless specified otherwise. HL cells (Washington Research Foundation, Seattle, USA) as well as *C. pneumoniae *(strain Kajaani*6, K6*) were kindly provided by Dr. P. Saikku (University of Oulu, Finland). Roquefort Societe (Société) was purchased from a general grocery supplier in Cambridge, United Kingdom. Cheese specimens were homogenized and processed for protein extraction before expiration dates. A/JSnYCit (A/Sn)/c mice, males aged from 2 to 4 months, were bred and kept under conventional conditions at the Animal Facilities of the Institute of Epidemiology and Microbiology (Moscow, Russia) in accordance with guidelines from the Russian Ministry of Health (number 755). Food and water were provided ad libitum. All experimental procedures were performed under a protocol approved by the Institutional Animal Care Committee.

### 2.2. Roquefort Fractionation

To obtain protein extracts a 10–15 g specimen of Roquefort cheese was placed in 10–15 mL of PBS and the samples were homogenized using an Omni TH-115. The resulting suspensions were kept for 1 hour at 4°C and centrifuged for 15 min at 10 000 g using an Eppendorf 5810R centrifuge. The obtained supernatant was centrifuged again for another 15 min at 10 000 g on an Eppendorf 5115D centrifuge. The resulting supernatant was used for further fractionation.

The protein extract was fractionated by gelfiltration on a 1.5 × 9.0 cm column with Sephadex G-25 Medium equilibrated with PBS. The column was precalibrated to determine free and total volume using Dextran Blue and DNP-L-Ala. For each experiment 3 mL of the cheese extract was introduced to the column and the elution fractions were collected as the following volumes: fraction number “1” as 6 mL, intermediate fraction “2” as 4 mL, and the last fraction “3” as 10 mL. Protein concentrations were determined by absorption at 280 nm on a Shimadzu UV-1.800 spectrophotometer. All three protein fractions were combined, dialyzed, lyophilized, and kept at −80°C for further studies.

### 2.3. *In Vitro* Studies

*C. pneumoniae *was initially propagated in HL cells and elementary bodies (EB) were purified by Renografin gradient centrifugation [15]. Chlamydial titers were determined by infecting HL cells with 10-fold dilutions of thawed stock suspension. Purified elementary bodies (EB) of known titer were suspended in sucrose-phosphate-glutamic acid buffer and used as inoculums for HL cells. Cells were grown in 24 well plates until a confluence rate of 80% was reached. HL plates were infected with *C. pneumoniae *at multiplicities of infection (MOI) of 1 in DMEM with FBS without cycloheximide and centrifuged for 1 hour at 1500 g. Addition of the cheese protein exract dissolved in PBS at concentrations of 0.12, 0.25, and 0.5 mg/mL was done simultaneously with the inoculation of *C. pneumoniae*.

#### 2.3.1. Immunofluorescence Staining

Infected HL monolayers grown on coverslips in 24-well plates for 72 hours were fixed with methanol. Permeabilized cells were stained by direct immunofluoresence (IF) using FITC—conjugated monoclonal antibody against chlamydial lipopolysaccharide (Nearmedic Plus, RF). Inclusion-containing cells were visualized using a Nikon Eclipse 50i fluorescence microscope at ×1350 magnification.

#### 2.3.2. Assessment of Infective Progeny

In order to assess the infective progeny accumulation in HL cells after a 72-hour cultivation period, HL cells were harvested, frozen, and thawed, as described elsewhere. Serial dilutions of lysates were inoculated onto HL cells and centrifuged for 1 hour at 1500 g. The infected cells were visualized with chlamydial LPS-specific monoclonal antibody after 72 hours of the postinfection period.

### 2.4. *In Vivo* LPS Stimulation

Mice were gavaged with 0.2 mL of PBS containing 10.0 mg of Roquefort cheese (once daily) or PBS alone. 24 hours after the last gavage procedure, control and cheese-fed mice were injected with 0.5 mg/kg LPS (*E. coli*, strain O111:B4) dissolved in PBS or PBS alone. Peritoneal washes were obtained 48 hours after LPS injection. Resulting cells were counted and recruited cell populations were analyzed by flow cytometry.

Peritoneal exudate cells were washed, resuspended in Fc block (Biolegend, USA) for 10 min and incubated with monoclonal antibodies (mAb) in FACS buffer (PBS, 2% FCS, and 0.05% sodium azide) for 15 min. Cells were then washed, resuspended, and analyzed using a FACS Calibur flow cytometer (BD Biosciences), CELLQUEST acquisition software (BD Biosciences), and FCS EXPRESS V.3 analysis software (DeNovo software, USA). The mAbs used were fluorescein-isothiocyanate- (FITC-) conjugated anti-Ly-6G (Myltenyi Biotech, Germany), RPE-conjugated anti-F4/80 (Invitrogen, USA), RPE-conjugated anti-CD11c (Invitrogen, USA)- fluorescein-isothiocyanate- (FITC-) conjugated anti-CD86 (Invitrogen, USA), RPE-conjugated anti-B220 (Invitrogen, USA), phycoerythrin- (PE-) conjugated anti-TCR (Biolegend, USA), fluorescein-isothiocyanate- (FITC-) conjugated anti-CD4 (Biolegend, USA), and PerCP-conjugated anti-CD8 (Biolegend, USA).

### 2.5. Statistical Analysis

All values are expressed as the mean ± SD. Variation between data sets was evaluated using Student's *t*-test. Changes with *P* values less than 0.05 were considered statistically significant. All experiments were repeated at least 3 times. Most representative IF images were chosen for publishing.

## 3. Results

**Figure 1:**
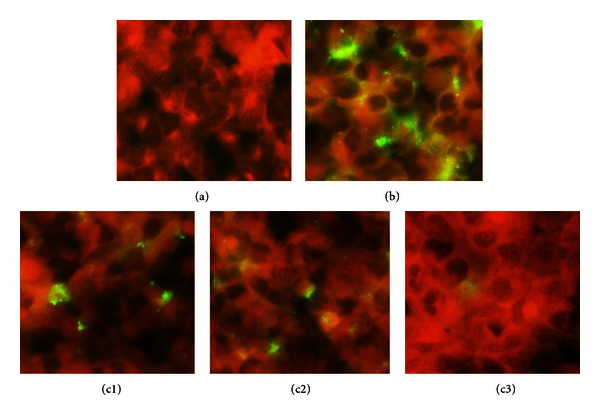
HL cells were plated, grown, and infected with *C. pneumoniae* at MOI 1. Additions of the Roquefort cheese protein extract with final concentrations of 0.12, 0.25, and 0.50 mg/mL were performed simultaneously with the bacterial pathogen inoculation. The cell monolayers were harvested after 72 h incubation at 35°C in 5% CO_2_ and immunofluorescent staining conducted as described in Section 2. Inhibition of *C*. *pneumonia* growth by roquefort protein extract ((a) uninfected cells; (b) infected cells; (c1), (c2), and (c3) infected cells grown in the presence of 0.12, 0.25, and 0.50 mg/mL Roquefort protein extract, resp.).

As can be seen from Figure 1, infecting HL cells with *C. pneumoniae* strain leads to the formation of typical, densely stained, round-shaped inclusions of different sizes inside the host cells. Addition of the Roquefort cheese protein extract led to dose-dependent inhibition of inclusion body formation with their complete disappearance at a concentration of 0.5 mg/mL. Lower concentrations of cheese extract (0.12 and 0.25 mg/mL) induced the formation of atypical pleomorphic inclusion bodies, which were generally smaller in size and poorly stained and seen in a smaller number of cells as compared to the control. Cytotoxicity assessment showed that incubation of the host cells with cheese extract alone was not accompanied by changes in cell growth and their viability (results not shown). The results obtained were in good agreement with the data revealing the effect of Roquefort cheese extract on *C. pneumoniae* infection progeny formation. Table 1 shows that Roquefort protein extract caused stepwise reduction in the infective progeny number. Interestingly, on multiple repeats, the highest concentration of the cheese extract used in our experiments did not cause complete eradication of infective progeny of *C. pneumoniae*.

**Figure 2:**
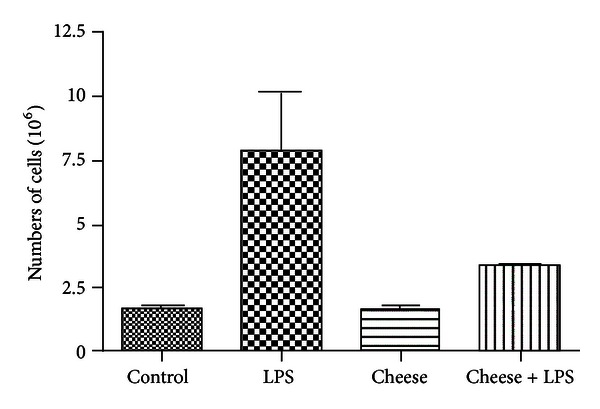
Peritoneal cell count in LPS-stimulated mice fed with Roquefort cheese. Mice were gavaged twice (once daily) with PBS containing 10.0 mg of blue-veined cheese or PBS alone (control). 24 hours later, the animals were injected intraperitoneally with LPS or its solvent (PBS). Peritoneal washes were obtained 48 hours after LPS injection and the resulting cell suspensions were counted as described in Section 2.

The development and outcomes of chlamydial infections are intrinsically predetermined by both the virulence of the pathogen and the ability of the host organism to develop an adequate physiological response to bacterial insult. Therefore, next, we tried to evaluate the ability of the Roquefort protein extract to affect an intraperitoneal cell migratory response induced by injection of LPS* in vivo*. As it can be seen from Figure 2, IP injection of LPS induces an exuberant cellular migratory response into the peritoneal cavity which was revealed by ~3.5-fold increase in the number of cells detectable in peritoneal washes. Although Roquefort feeding by itself did not affect the peritoneal cell count, IP injection of LPS into cheese-fed mice was accompanied by a significant attenuation of the peritoneal migratory response manifested by a smaller increase in the number of peritoneal cells.

**Figure 3:**
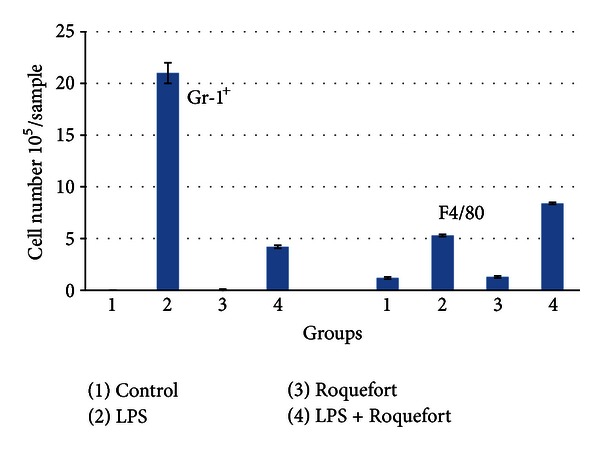
Neutrophil (Gr-1^+^) and macrophage (F4/80) counts in peritoneal washes obtained from LPS-stimulated mice fed with Roquefort cheese. Mice were gavaged twice (once daily) with PBS containing 10.0 mg of the Roquefort protein extract or PBS alone (control). 24 hours later, the animals were injected intraperitoneally with LPS or its solvent (PBS). Peritoneal washes were obtained 48 hours after LPS injection. Neutrophil (Gr-1^+^) and macrophage (F4/80) populations were quantified as described in Section 2.

Next we decided to analyze the subpopulations of the peritoneal cells recruited into the LPS-induced migratory response. First, we compared Gr-1^+^ (neutrophil) and F4/80^+^ (macrophage) populations as two major and crucial players in early inflammatory events. As shown in Figure 3, the number of neutrophils (Gr-1^+^) was extremely low in peritoneal washes obtained from intact mice. However, it was dramatically increased in the LPS stimulated mice. The neutrophil count after LPS injection was noticeably lower in the cheese-fed mice as compared to the group of mice with no cheese feeding. On the other hand, Roquefort feeding promoted accumulation of macrophages in the peritoneal washes upon LPS stimulation, whereas it had no effect on macrophage counts in the PBS-injected mice.

**Figure 4:**
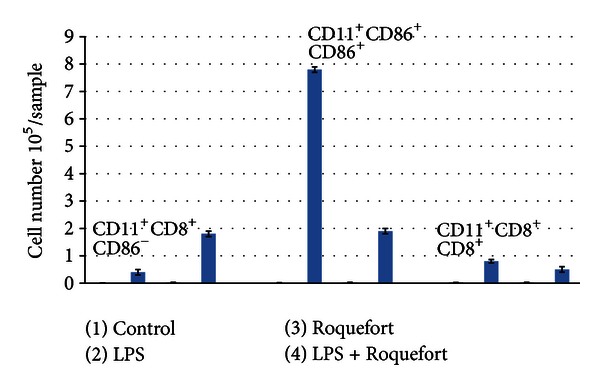
Dendritic cell subpopulations in peritoneal washes obtained from LPS-stimulated mice fed with Roquefort cheese. Mice were gavaged twice (once daily) with PBS containing 10.0 mg of Roquefort cheese or PBS alone (control). 24 hours later, the animals were injected intraperitoneally with LPS or its solvent (PBS). Peritoneal washes were obtained 48 hours after LPS injection. Dendritic cell subpopulations (CD11^+^CD8^+^CD86^−^, CD11^+^CD8^+^CD86^+^, and CD11^+^CD8^+^CD^+^8^+^) were quantified as described in Section 2.

In addition we analyzed CD11c^+^ dendritic cell subpopulations in the peritoneal washes obtained from LPS-stimulated mice. Depending on CD8 expression two major DC populations are traditionally referred to as myeloid (CD11c^+^CD8^−^) and plasmocytoid (CD11c^+^CD86^+^) dendritic cells [16]. As shown in Figure 4, LPS stimulation in the absence of Roquefort feeding results in a pronounced increase in myeloid dendritic cells expressing CD86. The level of this cell population in the cheese-fed mice was unchanged in comparison with control values. In contrast, the level of plasmocytoid dendritic cells expressing CD86 was higher in the Roquefort-fed LPS-treated mice as compared to LPS-treated mice and control group.

## 4. Discussion

*C. pneumoniae* is an airborne obligate bacterial pathogen responsible for a significant number of respiratory infections around the world and implemented in the pathogenesis of atherosclerosis. Unlike other Gram-negative bacteria, *C. pneumoniae* has a unique biology and is capable of completing its life cycle inside host cells. The intracellular location of the pathogen as well as its ability to infect cells with migratory potential (mononuclear leukocytes and lymphocytes) promotes the generalization of *C. pneumoniae *infection in the human body and creates a significant challenge for pharmacotherapy [17]. There are multiple reports regarding identification of *C. pneumoniae *in the tissues of the cardiovascular system, joints, brain, and meninges [18]. These observations raise a valid question about the etiological significance of *C. pneumoniae* in atherosclerosis, arthritis, and some neurological diseases.

Our major finding reported in this paper is that a protein fraction obtained from Roquefort cheese exhibits significant inhibitory activity on *C. pneumoniae* growth in host cells. This observation has been made and repeated multiple times in our experiments using a classical immunofluorescence assay in U-cells as well as measurement of the infective progeny formation in HL cells. Despite our confidence in the observed phenomenon, it has to be yet determined which particular component/s is/are responsible for the antichlamydial activity of the Roquefort cheese. The cheese core of ripened moulded cheeses contains a unique variety of substances of mammalian, bacterial and fungal origin [19]. Interestingly, the genome of *Penicillium roqueforti*, a major fungus used for Roquefort production, does not contain genes encoding penicillin biosynthesis [20]. Some other substances, in particular roquefortine, are believed to have antibacterial activity [14]. It is also important to mention that in our preliminary studies a crude Roquefort cheese homogenate had noticeable cytotoxicity which was attributable, in our opinion, to the presence of organic substances of low molecular weight. This activity became redundant after size-exclusion chromatography and subsequent dialysis of the resulting fractions. Indeed, Roquefort cheese has been shown to contain a wide range of organic compounds with potential cytotoxicity—mycophenolic acid, putrescine, tyramine, and others [11]. Only purified protein fraction of Roquefort cheese were found in our experiments to have reproducible and dose-dependent antichlamydial activity. It has to be determined in future whether our *in vitro *observation has any possible implication for *Chlamydia* infection *in vivo* conditions. Animal studies, and possibly clinical trials, will be required to assess this potential.

Besides antichlamydial activity, Roquefort cheese proteins have a noticeable impact on the mechanisms of innate immunity. Using an *in vivo *LPS stimulation model we have demonstrated that oral supplementation with Roquefort attenuated the overall peritoneal migratory response. The attenuation of the cell migratory response has clearly occurred as a result of the reduced neutrophil influx, while the macrophage number in the peritoneal washes of LPS-stimulated mice fed with the cheese-derived proteins was consistently raised. It is worthwhile mentioning that attenuated cell migratory response of neutrophils is generally believed to lessen tissue damage during subsequent release of inflammatory mediators, whereas macrophage influx promotes reparative events at the site of inflammation [21].

In addition we observed that the Roquefort feeding can modulate the dendritic cell subsets *in vivo* conditions. A possible role of these cells in the development of atherosclerosis has been of a particular interest. For example, it has been shown that statins can exert their ability to inhibit atherosclerosis by affecting the dendritic cell subpopulations [16]. In our study Roquefort cheese feeding decreased the recruitment of “myeloid” dendritic cells, while some increase in recruitment was observed for plasmacytoid dendritic cells. The exact beneficial or pathogenic consequences of these differential activities of Roquefort cheese on dendritic cells need further elucidation.

Although the identification of proteins responsible for Roquefort antichlamydial activity and the exact mechanisms of their actions remain unclear, our results represent the first evidence demonstrating an antichlamydia activity associated with Roquefort cheese. It is tempting to assume that systematic dietary intake of fungal fermented cheeses, as a key element of French gastronomic tradition, may have some positive impact on the level of the control of this infection and associated inflammatory conditions, and consequently on the prevalence of cardiovascular disease in this country. Although this assumption is highly speculative, there is epidemiological evidence suggesting that Southern France, a region historically associated with high Roquefort cheese production and consumption, has remarkably low rates of cardiovascular mortality.

## Figures and Tables

**Table 1:** HL cells were plated, grown, and infected with *C. pneumoniae* at MOI 1. Additions of the Roquefort cheese extract fraction with final concentrations of 0.12, 0.25, and 0.55 mg/mL were performed simultaneously with the bacterial pathogen inoculation. The cell monolayers were harvested after 72 hours incubation at 35°C in 5% CO_2_ and infective progeny number was determined as described in Section 2. Infective progeny formation in U-937 cells infected with *C. pneumoniae* in the presence of cheese protein extract.

Cheese extract (mg/mL)	Infective progeny IFU/mL
0	2.4 × 10^5^ ± 1.3
0.12	2.8 × 10^4^ ± 0.9*
0.25	7.2 × 10^2^ ± 2.1*
0.50	5.8 × 10^1^ ± 2.7*

*Significant changes as compared with control, *P* ≤ 0.05.
